# Patterns of coronary heart disease mortality over the 20^th ^century in England and Wales: Possible plateaus in the rate of decline

**DOI:** 10.1186/1471-2458-8-148

**Published:** 2008-05-01

**Authors:** Steven Allender, Peter Scarborough, Martin O'Flaherty, Simon Capewell

**Affiliations:** 1 Division of Public Health and Primary Health Care, University of Oxford, Oxford, UK; 2 Division of Public Health, University of Liverpool, UK

## Abstract

**Background:**

Coronary heart disease (CHD) rates in England and Wales between 1950 and 2005 were high and reasonably steady until the mid 1970s, when they began to fall. Recent work suggests that the rate of change in some groups has begun to decrease and may be starting to plateau or even reverse.

**Methods:**

Data for all deaths between 1931 and 2005 in England and Wales were grouped by year, sex, age at death and contemporaneous ICD code for CHD as cause of death. CHD mortality rates by calendar year and birth cohort were produced for both sexes and rates of change were examined.

**Results:**

The pattern of increased burden of CHD mortality within older age groups has only recently emerged in men, whereas it has been established in women for far longer. CHD mortality rates among younger people showed little variation by birth cohort. For younger women (49 and under), the rate of change in CHD mortality has reversed in the last 20 years, indicating a future plateau and possible reversal of previous improvement in CHD mortality rates. Among younger men the rate of change in CHD mortality has been consistent for the past 15 years indicating that rates in this group have continued to fall steadily.

**Conclusion:**

Although CHD mortality rates continue to drop in older age groups the actual burden of coronary heart disease is increasing due to the ageing of the population. The rate of improvement in CHD mortality appears to be beginning to decline and may even be reversing among younger women.

## Background

Cardiovascular disease appears set to continue as the dominant cause of death and disability in the UK. Age-standardised coronary heart disease (CHD) rates in England and Wales were high between 1950 and the mid 1970s. After this time CHD mortality rates began to fall and have been falling since [1]. Age-standardisation of CHD mortality rates allows for direct comparison between different time periods, when the age structure of England and Wales were significantly different. However, little work has been done on long term trends in age-stratified CHD mortality rates. One recent study suggested a period effect: that patterns in CHD mortality rates between 1920 and 1986 were similar for diverse age groups and, while rates were higher in higher age groups, all age groups experienced a peak in the mid 1970s [2]. Closer examination of age-stratified rates is very important because, when only age-standardised rates are considered, reductions in the mortality rate in older age groups may obscure less positive trends in younger men and women.

The recent trends in cardiovascular risk factor prevalence in younger people are disturbing. Obesity levels in people aged 45 and under have been rising for over ten years, and there has been a similar increase in type 2 diabetes. Physical activity levels in younger people decreased slightly over the last fifteen years, and smoking levels within this age group have remained high, with approximately 25% of men and 20% of women under 45 smoking [3]. These adverse trends could begin to slow, or even reverse, the previously observed decline in mortality rates within younger age groups. Evidence from Australia [4] and America [5] suggests some levelling out of CHD mortality rates among younger men and women and warn that CHD mortality rates in these groups may be starting to increase.

Studying the patterns of mortality over time for chronic disease is important. Firstly, to assess whether current disease reduction strategies are proving to be successful. Secondly, to allow inferences to be made about the contribution of different population factors to disease risk. It is useful to examine long term trends as this allows for recent trends to be viewed in perspective as well as providing a history of the disease burden. As well as recording age-stratified mortality rates over time, it is also useful to record patterns in the number of CHD deaths experienced by different age groups. Such patterns allow for an estimate of the burden of CHD that incorporates the dynamics of an ageing population which are otherwise obscured by declining mortality rates.

A useful alternative to recording trends by calendar year is to examine the experiences of different birth cohorts. This approach allows for a direct comparison of CHD mortality rates within different generations. Such a method can be used to compare the effect of environmental exposures over the whole life course, or those which are typical for a given generation [6].

Recording long-term CHD mortality trends requires use of different definitions for CHD drawn from different International Classification of Diseases (ICD) revisions. Inconsistencies in definitions between revisions effectively limits the potential time period under consideration; it is generally agreed that reliable coding of CHD did not begin until the fourth ICD revision (in 1931) [7]. Reasonably consistent definitions have existed since the seventh ICD revision in 1967 [8]. Because of discontinuities in trends due to definition revisions the patterns in CHD mortality from the first half of the twentieth century should be treated with caution.

This paper therefore examines the variation in CHD mortality by calendar year and by birth cohort for all registered deaths between 1931 and 2005 in England and Wales with a particular focus on recent trends for the younger age groups.

## Methods

Data on all deaths in England and Wales in the twentieth century by year, sex, age at death and contemporaneous ICD code for cause of death were provided by the Office for National Statistics, along with population estimates for each year-age-sex group. These data were accompanied by data on population estimates, and the number of CHD deaths by age and sex for 2001 to 2005. The data used in this study were subject to previous ethical review within the Office for National Statistics. Separate ethics approval was not considered necessary for this analysis.

A review of literature considering long term trends in CHD produced a framework for the coding of CHD over different revisions of the ICD [6,7,9,10]. Where there was ambiguity over ICD codes one of the authors (SC), a researcher with extensive experience in heart disease, provided a final decision on CHD codes. A coding frame for CHD incorporating ICD revisions 4 to 10 was constructed and is shown in Table 1. This coding frame was used to reduce the twentieth century data to deaths from CHD since 1931.

**Table 1:** Coding frame for coronary heart disease, 1931 to 2005

**ICD revision**	**ICD code**	**Primary description**	**Secondary description**
ICD-4	94	Diseases of the coronary arteries, Angina pectoris	
ICD-5	94b	Angina pectoris without mention of coronary disease	
ICD-5	94a	Diseases of the coronary arteries	
ICD-6	4202	Arteriosclerotic heart disease, including coronary disease	Angina pectoris without mention of coronary disease
ICD-6	4201	Arteriosclerotic heart disease, including coronary disease	Heart disease specified as involving coronary arteries
ICD-6	4200	Arteriosclerotic heart disease, including coronary disease	Arteriosclerotic heart disease so described
ICD-7	4202	Arteriosclerotic heart disease, including coronary disease	Angina pectoris without mention of coronary disease
ICD-7	4201	Arteriosclerotic heart disease, including coronary disease	Heart disease specified as involving coronary arteries
ICD-7	4200	Arteriosclerotic heart disease, including coronary disease	Artetriosclerotic heart disease so described
ICD-8	4149	Asymptomatic ischaemic heart disease	Without mention of hypertensive disease
ICD-8	4140	Asymptomatic ischaemic heart disease	With hypertensive disease
ICD-8	4139	Angina pectoris	Without mention of hypertensive disease
ICD-8	4130	Angina pectoris	With hypertensive disease
ICD-8	4129	Chronic ischaemic heart disease	Other endocardial structures
ICD-8	4124	Chronic ischaemic heart disease	Cardiovascular disease without mention of hypertension or chronic ischaemic heart disease
ICD-8	4123	Chronic ischaemic heart disease	Chronic ischaemic heart disease with no mention of hypertension
ICD-8	4122	Chronic ischaemic heart disease	Cardiovascular disease with hypertension but no mention of chronic ischaemic heart disease
ICD-8	4121	Chronic ischaemic heart disease	Chronic ischaemic heart disease with hypertension
ICD-8	4120	Chronic ischaemic heart disease	Chronic ischaemic heart disease
ICD-8	4119	Other acute and sub-acute forms of ischaemic heart disease	Without mention of hypertensive disease
ICD-8	4110	Other acute and sub-acute forms of ischaemic heart disease	With hypertensive disease
ICD-8	4109	Acute myocardial infarction	Without mention of hypertensive disease
ICD-8	4100	Acute myocardial infarction	With hypertensive disease
ICD-9	4149	Other forms of chronic ischaemic heart disease	Unspecified
ICD-9	4148	Other forms of chronic ischaemic heart disease	Other
ICD-9	4141	Other forms of chronic ischaemic heart disease	Aneurysm of heart
ICD-9	4140	Other forms of chronic ischaemic heart disease	Coronary atherosclerosis
ICD-9	4130	Angina pectoris	Angina pectoris
ICD-9	4120	Old myocardial infarction	Old myocardial infarction
ICD-9	4110	Other acute and subacute forms of ischaemic heart disease	Other acute and subacute forms of ischaemic heart disease
ICD-9	4100	Acute myocardial infarction	Acute myocardial infarction
ICD-10	I20	Angina pectoris	
ICD-10	I21	Acute myocardial infarction (AMI)	
ICD-10	I22	Subsequent myocardial infarction	
ICD-10	I23	Certain current complications following AMI	
ICD-10	I24	Other acute ischaemic diseases	
ICD-10	I25	Chronic ischaemic heart disease	

The age-stratified number of CHD deaths, and the age-stratified CHD mortality rate (number of deaths/population) for men and women by calendar year were tabulated and graphed. The rate of change in CHD mortality rates was tracked by calculating the percentage difference between two sequential years, using moving five year averages for smoothing. The rate of change was tabulated and graphed by calendar year.

Ten year birth cohorts were constructed by subtracting the mean age of each five year age band from the year of death. The population for each birth cohort was derived within gender and age groups from annual population figures. Age-stratified CHD mortality rates were estimated as the number of CHD deaths within these groups, divided by the population. Confidence intervals for rates were calculated using the method described by Altman [11]. It should be noted that mortality rates for ages 30–34 and 35–39 within the 1895–1904 birth cohort were calculated using CHD mortality deaths that occurred from 1931 onwards. Age-stratified population levels for this cohort were calculated in a similar way as for other birth cohorts.

## Results

**Figure 1:**
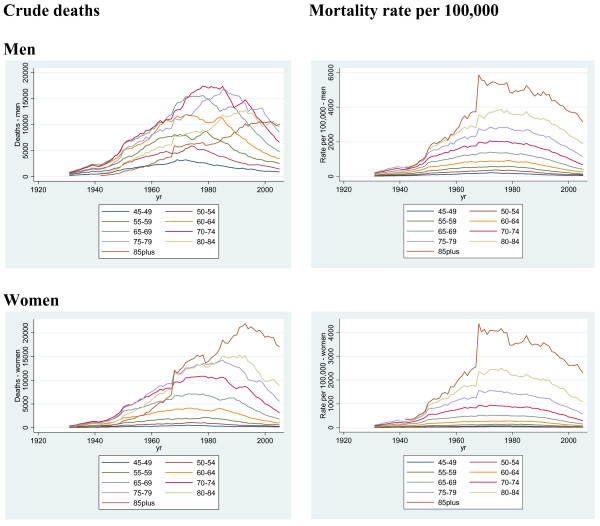
Crude CHD death rates and mortality rate per 100,000, by age group (45 and older), men and women, 1931 to 2005.

Age-stratified CHD mortality rates for men and women by calendar year are shown in Figure 1. They show a consistent pattern, both within age groups and gender, of a sharp increase until the mid 1970s, and then a steady decline. A sharp rise in the CHD mortality rate for both men and women is evident in 1967, the first year of the ICD-7 revision.

Figure 1 also shows the number of CHD deaths in older age groups of men and women by calendar year. The pattern of a higher burden of CHD mortality for older age groups has only recently emerged in men, whereas it has been established in women for far longer. As an example the CHD death rate among women aged 80–84 exceeded that for women age 60–64 for the first time in 1949, a phenomenon which did not occur in the same age groups in men until 1985 (Table 2).

**Table 2:** Coronary heart disease deaths, mortality rate, rate ratio and 95% confidence intervals for rate ratios by sex, age and birth cohort

**Men**	**30–34**	**35–39**	**40–44**	**45–49**	**50–54**	**55–59**	**60–64**	**65–69**
**Deaths**								
1895–1904	162	851	3113	10759	28186	55648	92238	127362
1905–1914	306	1757	7127	19881	43720	77885	115431	150718
1915–1924	955	3943	11902	28017	51781	78397	106744	128117
1925–1934	1461	5170	13436	26591	43228	58544	72621	81940
								
**Population (000s)**								
1895–1904	1669	1566	1598	1587	1524	1435	1292	1091
1905–1914	1683	1693	1688	1626	1562	1480	1337	1142
1915–1924	1728	1705	1696	1657	1602	1511	1383	1214
1925–1934	1533	1545	1523	1490	1436	1364	1259	1117
								
**Mortality rate per 100,000**								
1895–1904	10	54	195	678	1850	3877	7139	11670
1905–1914	18	104	422	1223	2799	5264	8634	13201
1915–1924	55	231	702	1690	3232	5190	7719	10557
1925–1934	95	335	882	1785	3011	4293	5770	7334
								
**95% CI mortality rate per 100,000**								
1895–1904	(8, 11)	(51, 58)	(188, 202)	(665, 691)	(1828, 1872)	(3846, 3909)	(7094, 7183)	(11609, 11730)
1905–1914	(16, 20)	(99, 109)	(412, 432)	(1206, 1240)	(2773, 2825)	(5228, 5300)	(8586, 8681)	(13139, 13263)
1915–1924	(52, 59)	(224, 239)	(689, 714)	(1671, 1710)	(3205, 3260)	(5155, 5226)	(7675, 7764)	(10502, 10611)
1925–1934	(90, 100)	(326, 344)	(867, 897)	(1764, 1806)	(2983, 3039)	(4259, 4327)	(5730, 5811)	(7285, 7382)
								
**Rate ratio**								
1895–1904	1	1	1	1	1	1	1	1
1905–1914	1.9	1.9	2.2	1.8	1.5	1.4	1.2	1.1
1915–1924	5.7	4.3	3.6	2.5	1.7	1.3	1.1	0.9
1925–1934	9.8	6.2	4.5	2.6	1.6	1.1	0.8	0.6
								
**95% CI rate ratio**								
1895–1904	-	-	-	-	-	-	-	-
1905–1914	(1.7, 2.1)	(1.8, 2.0)	(2.1, 2.2)	(1.8, 1.8)	(1.5, 1.5)	(1.3, 1.4)	(1.2, 1.2)	(1.1, 1.1)
1915–1924	(5.3, 6.1)	(4.1, 4.4)	(3.5, 3.7)	(2.5, 2.5)	(1.7, 1.8)	(1.3, 1.3)	(1.1, 1.1)	(0.9, 0.9)
1925–1934	(9.3, 10.3)	(6.0, 6.3)	(4.5, 4.6)	(2.6, 2.7)	(1.6, 1.6)	(1.1, 1.1)	(0.8, 0.8)	(0.6, 0.6)
								
**Women**	**30–34**	**35–39**	**40–44**	**45–49**	**50–54**	**55–59**	**60–64**	**65–69**
**Deaths**								
1895–1904	66	264	615	1938	5513	14124	32942	62900
1905–1914	110	317	1006	3082	7750	18683	39662	69061
1915–1924	158	485	1700	4250	9835	20067	37440	59255
1925–1934	233	741	2180	4437	8624	15657	25687	35007
								
**Population (000s)**								
1895–1904	1739	1709	1669	1648	1607	1554	1480	1366
1905–1914	1773	1750	1710	1670	1640	1595	1516	1394
1915–1924	1756	1752	1722	1702	1665	1609	1534	1430
1925–1934	1541	1539	1527	1503	1466	1419	1359	1270
								
**Mortality rate per 100,000**								
1895–1904	4	15	37	118	343	909	2226	4603
1905–1914	6	18	59	185	473	1172	2616	4953
1915–1924	9	28	99	250	591	1247	2441	4143
1925–1934	15	48	143	295	588	1103	1890	2757
								
**95% CI mortality rate per 100,000**								
1895–1904	(3, 5)	(14, 17)	(34, 40)	(112, 123)	(334, 352)	(894, 924)	(2203, 2250)	(4568, 4639)
1905–1914	(5, 7)	(16, 20)	(55, 63)	(178, 191)	(462, 483)	(1155, 1188)	(2590, 2641)	(4917, 4989)
1915–1924	(8, 10)	(25, 30)	(94, 103)	(242, 257)	(579, 603)	(1230, 1264)	(2417, 2466)	(4110, 4176)
1925–1934	(13, 17)	(45, 52)	(137, 149)	(287, 304)	(576, 601)	(1086, 1120)	(1868, 1913)	(2729, 2786)
								
**Rate ratio**								
1895–1904	1	1	1	1	1	1	1	1
1905–1914	1.6	1.2	1.6	1.6	1.4	1.3	1.2	1.1
1915–1924	2.4	1.8	2.7	2.1	1.7	1.4	1.1	0.9
1925–1934	4.0	3.1	3.9	2.5	1.7	1.2	0.8	0.6
								
**95% CI rate ratio**								
1895–1904	-	-	-	-	-	-	-	-
1905–1914	(1.3, 2.0)	(1.0, 1.3)	(1.5, 1.7)	(1.5, 1.6)	(1.3, 1.4)	(1.3, 1.3)	(1.2, 1.2)	(1.1, 1.1)
1915–1924	(2.0, 2.8)	(1.6, 2.0)	(2.6, 2.8)	(2.1, 2.2)	(1.7, 1.8)	(1.4, 1.4)	(1.1, 1.1)	(0.9, 0.9)
1925–1934	(3.5, 4.5)	(2.9, 3.4)	(3.7, 4.0)	(2.4, 2.6)	(1.7, 1.8)	(1.2, 1.2)	(0.8, 0.9)	(0.6, 0.6)

* Base for rate ratio is the age-stratified mortality rate for the 1895–1904 cohort.

† 95% confidence intervals constructed assuming Poisson distribution of death count variable (Altman, 2000)

In 1945 there were 1,823 and 178 less CHD deaths in the older age group (aged 80 to 84) than in the younger age group (aged 60 to 64) among men and women respectively. Ten years later, in 1955, the older age group among women experienced 1,608 **more **CHD deaths than the younger age group compared with 2,409 **less **CHD deaths among men. Another fifty years on in 2005 there were 6,114 and 7,839 more CHD deaths in the older age group among men and women respectively.

**Figure 2:**
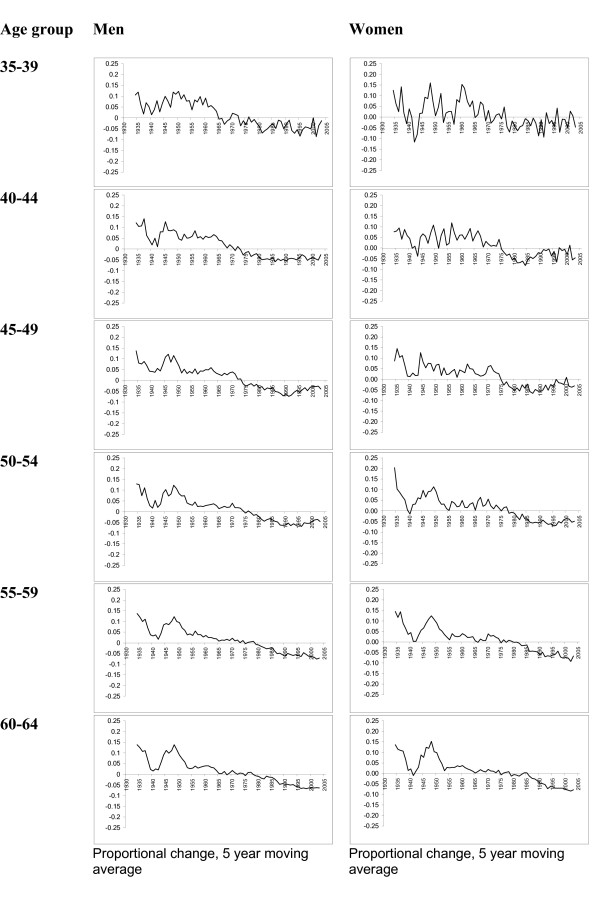
Change in CHD mortality rate per 100,000, by age group (35 to 64), men and women, 1931 to 2005.

**Figure 3:**
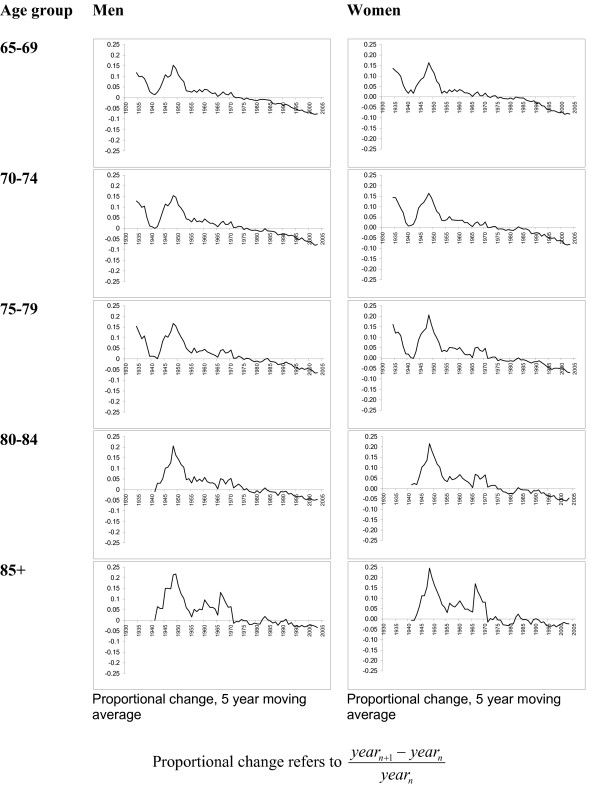
Change in CHD mortality rate per 100,000, by age group (65 and older), men and women, 1931 to 2005.

The rate of change of CHD mortality rates for each age-sex group is shown in Figures 2 and 3. For younger women (under 50), the rate of change in CHD mortality has reversed in the last 20 years. The line in these charts crossing zero suggests a future plateau and possible reversal of previous improvement in CHD mortality rates. Among younger men, the rate of change in CHD mortality has been more consistent than for women over the past 15 years. The line in these charts remaining below zero indicates that CHD mortality rates in this group have continued to fall steadily. In men aged 45 to 49 and 50 to 54 there appears to be a small reverse in the most recent years included in this series. This pattern is very different within older people where the rate of change in CHD mortality rates has continued to improve over the past 20 years.

**Figure 4:**
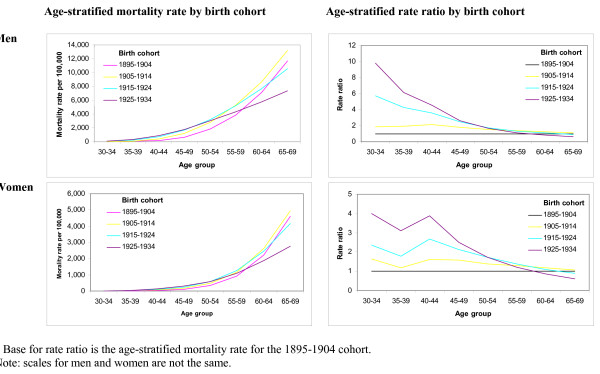
Age-stratified (30 to 69 year olds) mortality rates and standardised mortality ratios by birth cohort.

Variations in CHD mortality rates between those birth cohorts with deaths up to age 59 shows that for both men and women the cohort born in 1925 to 1934 experienced higher rates of coronary heart disease than the 1895 to 1904 cohort (Figures 2, 3 and 4). This trend was observed for all ages from 30–34 to 55–59. As an example of one extreme the CHD rate ratio for 1925 – 1934 cohort using the 1895–1904 cohort as a reference shows that among younger men the rate was as much as ten times higher than the earlier cohort (for men aged 30–34) and four times higher for women (aged 30–34). Rate ratios (and 95% confidence intervals) suggest that these comparisons represent significant differences in the rates between the two time periods. Similarly the rate ratio (and 95% CIs) for those aged 60 and over for both men and women are less than 1.0 suggesting that the trend is for lower mortality rates in older birth cohorts. Table 3 presents mortality rates and rate ratios for ten year time periods. Among men in the youngest age groups the rate ratio comparing CHD mortality in recent calendar years with the referent time period (1936–45) shows that CHD mortality rates were consistently higher in all but the oldest male age group. The rate ratios for women suggest that the progress in relative reduction of CHD mortality rates has been more consistent over time.

**Table 3:** Coronary heart disease deaths, mortality rate, rate ratio and 95% confidence intervals for rate ratios by sex, age and year of death

**Men**	**30–34**	**35–39**	**40–44**	**45–49**	**50–54**	**55–59**	**60–64**	**65–69**
**Deaths**								
1936–45	273	936	2561	5347	10076	15752	21094	23970
1946–55	810	2291	6376	13949	24001	34165	47520	57763
1956–65	1396	4783	10782	21895	41820	65473	83693	92738
1966–75	1576	4946	13834	29887	50471	77214	114210	141632
1976–85	1520	4524	10966	24027	46480	77437	109151	143514
1986–95	1051	3333	8248	16436	28719	49653	81613	119728
1996–2005	810	2332	5550	10987	19740	30404	45011	66886
								
**Mortality rate per 100,000**								
1936–45	2	6	19	42	87	147	226	311
1946–55	5	14	38	86	163	289	492	736
1956–65	9	28	64	135	268	443	662	1040
1966–75	10	33	87	180	315	522	854	1251
1976–85	8	25	72	167	311	513	789	1257
1986–95	5	18	45	91	195	374	627	987
1996–2005	4	11	28	61	111	177	334	577
								
**Rate ratio**								
1936–45	1.0	1.0	1.0	1.0	1.0	1.0	1.0	1.0
1946–55	2.9	2.3	2.0	2.0	1.9	2.0	2.2	2.4
1956–65	5.4	4.7	3.4	3.2	3.1	3.0	2.9	3.3
1966–75	6.2	5.5	4.6	4.3	3.6	3.6	3.8	4.0
1976–85	5.0	4.1	3.8	3.9	3.6	3.5	3.5	4.0
1986–95	3.2	3.0	2.4	2.1	2.2	2.5	2.8	3.2
1996–2005	2.4	1.9	1.5	1.4	1.3	1.2	1.5	1.9
								
**95% CI rate ratio**								
1936–45	-	-	-	-	-	-	-	-
1946–55	(2.7, 3.1)	(2.2, 2.4)	(1.9, 2.0)	(2.0, 2.1)	(1.8, 1.9)	(1.9, 2.0)	(2.2, 2.2)	(2.3, 2.4)
1956–65	(5.1, 5.6)	(4.6, 4.8)	(3.3, 3.4)	(3.1, 3.2)	(3.1, 3.1)	(3.0, 3.0)	(2.9, 2.9)	(3.3, 3.4)
1966–75	(5.9, 6.5)	(5.4, 5.7)	(4.5, 4.7)	(4.2, 4.3)	(3.6, 3.7)	(3.5, 3.6)	(3.8, 3.8)	(4.0, 4.0)
1976–85	(4.8, 5.3)	(4.0, 4.2)	(3.7, 3.9)	(3.9, 4.0)	(3.5, 3.6)	(3.5, 3.5)	(3.5, 3.5)	(4.0, 4.1)
1986–95	(3.0, 3.4)	(2.9, 3.1)	(2.3, 2.4)	(2.1, 2.2)	(2.2, 2.3)	(2.5, 2.6)	(2.8, 2.8)	(3.2, 3.2)
1996–2005	(2.3, 2.6)	(1.8, 2.0)	(1.4, 1.5)	(1.4, 1.5)	(1.3, 1.3)	(1.2, 1.2)	(1.5, 1.5)	(1.8, 1.9)
								
**Women**	**30–34**	**35–39**	**40–44**	**45–49**	**50–54**	**55–59**	**60–64**	**65–69**
								
**Deaths**								
1936–45	111	267	541	1199	2452	4733	8301	12243
1946–55	133	371	924	2351	4911	9884	19053	31573
1956–65	226	574	1542	3348	7360	15934	30830	50826
1966–75	275	762	2147	4666	9469	18883	39113	67127
1976–85	312	711	1753	3935	9260	20032	37935	65485
1986–95	247	575	1349	2730	5744	13102	29180	55030
1996–2005	190	518	1134	2275	4013	7619	14485	27489
								
**Mortality rate per 100,000**								
1936–45	0.6	1.5	3.3	7.9	17.4	36.5	71.4	126.0
1946–55	0.8	2.1	5.4	14.1	31.2	69.8	152.1	291.8
1956–65	1.4	3.3	9.0	20.0	44.9	100.0	211.7	412.9
1966–75	1.8	5.2	13.4	27.4	56.9	118.4	258.0	481.4
1976–85	1.7	3.9	11.7	27.3	60.2	124.5	247.3	469.7
1986–95	1.2	3.1	7.3	15.0	38.8	95.2	205.2	384.7
1996–2005	0.9	2.5	5.5	12.5	22.3	43.3	102.7	222.0
								
**Rate ratio**								
1936–45	1.0	1.0	1.0	1.0	1.0	1.0	1.0	1.0
1946–55	1.2	1.4	1.6	1.8	1.8	1.9	2.1	2.3
1956–65	2.2	2.1	2.7	2.5	2.6	2.7	3.0	3.3
1966–75	2.9	3.4	4.1	3.5	3.3	3.2	3.6	3.8
1976–85	2.7	2.5	3.6	3.5	3.5	3.4	3.5	3.7
1986–95	1.9	2.0	2.2	1.9	2.2	2.6	2.9	3.1
1996–2005	1.5	1.6	1.7	1.6	1.3	1.2	1.4	1.8
								
**95% CI rate ratio**								
1936–45	-	-	-	-	-	-	-	-
1946–55	(1.0, 1.4)	(1.2, 1.5)	(1.5, 1.8)	(1.7, 1.9)	(1.7, 1.8)	(1.9, 2.0)	(2.1, 2.2)	(2.3, 2.3)
1956–65	(1.9, 2.5)	(2.0, 2.3)	(2.6, 2.9)	(2.5, 2.6)	(2.5, 2.6)	(2.7, 2.8)	(2.9, 3.0)	(3.2, 3.3)
1966–75	(2.6, 3.2)	(3.1, 3.6)	(3.9, 4.3)	(3.4, 3.6)	(3.2, 3.3)	(3.2, 3.3)	(3.6, 3.6)	(3.8, 3.9)
1976–85	(2.4, 3.0)	(2.3, 2.7)	(3.4, 3.7)	(3.4, 3.6)	(3.4, 3.5)	(3.4, 3.5)	(3.4, 3.5)	(3.7, 3.8)
1986–95	(1.7, 2.2)	(1.9, 2.2)	(2.1, 2.4)	(1.8, 2.0)	(2.2, 2.3)	(2.6, 2.7)	(2.8, 2.9)	(3.0, 3.1)
1996–2005	(1.3, 1.7)	(1.5, 1.8)	(1.6, 1.8)	(1.5, 1.6)	(1.2, 1.3)	(1.2, 1.2)	(1.4, 1.5)	(1.7, 1.8)

## Discussion

In this paper we set out to examine the variation in CHD mortality by calendar year and birth cohort for all registered deaths in England and Wales between 1931 and 2005, a longer time period than previous studies.

### Summary of main findings

We found evidence of a higher burden of CHD mortality for older age groups, which has only recently emerged in men, whereas it has been established in women for much longer. We also observed a previously reported peak [1] in the mortality rate among most age groups in the late seventies followed by a more recent decline.

We found a plateau in the CHD mortality rate among younger age groups for women, a trend which was not observed in men. Recent work has suggested that CHD mortality rates in younger men and women are heading for a plateau, based on an estimation of the average annual change in mortality rates since 1984, and probably reversing [10,12]. The results presented here support this conclusion for young women (49 and younger), where the rate of change of CHD mortality rates appears to be converging on zero. The case in men is more complex: in younger men the rate of change of CHD mortality rates appears to have stabilized at a level below zero, suggesting that the current speed of decline has levelled out, but CHD mortality rates are still falling. In men between 45 and 54 years, there appears to be a small decrease which is less evident as in women. For older age groups of both men and women, the speed of reduction in CHD mortality rates continues to increase.

We observed that CHD mortality among younger age groups has increased in those born in the early twentieth century compared to those born in the late 19^th ^century. This requires further study as the public health implications of a decline in survival from CHD in younger age groups may be stark. This pattern suggests that although significant advances have been made among older populations similar gains are not being made in those less than 60 years of age. There are a number of possible reasons for this including the targeting and efficacy of screening, the inclusion criteria for beginning of treatment regimes (which include age as a standard risk factor), and the current public health focus on mortality reduction in older populations. The trends in CHD mortality rates by age group and birth cohort show how more recent generations have enjoyed far lower CHD mortality rates than those born in the late 19^th ^and early 20^th ^centuries.

### Strengths and Limitations

The paper presents age stratified CHD mortality rates over a long time period and contributes to the debate over the pattern of CHD mortality among younger age groups. It also provides an analysis of mortality trends across this time period within birth cohorts. It should be noted that comparisons between birth cohorts at the same age should be made with caution, as the numerators of the rates (number of CHD deaths) are based on slightly different definitions of CHD.

The reduction of the initial data to deaths from 1931 onwards is likely to have some effect on rates calculated for those born in 1895–1904 at ages 27–36. Age-stratified population levels for this cohort were calculated in a similar way as for other birth cohorts but there is likely to be an under-estimate of mortality rates in these groups. The possible under-estimate is in the order of 2% and should be borne in mind when interpreting results.

Any study using mortality data across multiple revisions of the International Statistical Classification of Diseases (ICD) will suffer attribution bias due to both the change between versions of ICD and the procedures to code deaths. In coding the data set within each ICD revision to a 'coronary heart disease' summary variable we have attempted to include underlying and contributing CHD mortality. In coding ICD-4, for example, angina pectoris both specified to CHD and without mention of CHD were coded to CHD in the parent data set (Table 1). This approach should be inclusive of all CHD over the time period. Differences across revisions of ICD also have the potential to affect the coding of CHD mortality. Deaths coded in versions of ICD prior to ICD-4 were considered so disparate as to be incomparable over time. For this reason the analysis here deals with deaths coded in 1931 onwards.

Jannsen and Kunst [8] examined the changes in deaths around ICD coding changes and found some evidence that ischaemic heart disease was affected. Their findings are difficult to interpret in relation to this paper as other major causes of mortality around changes in the coding such as floods or World Wars may also create unusual patterns in the data. Further, Jannsen and Kunst suggest that outliers may account for much of the observed differences in trend.

The change in ICD coding in the transition from ICD-9 to ICD-10 represents a large shift in disease coding [1] and for this reason comparisons between the last ten year period of deaths (1996–2005) and previous revisions should be interpreted with caution. In some other studies a "correction factor" is applied to adjust for differences in coding across different revisions of ICD. These correction factors, published by the UK Office for National Statistics among others, are useful for understanding trends around the transition period for ICD codes. They are not recommended for use in interpreting trend data over many revisions of ICD [1] and are not used here.

Changes in methods of death certification create a potential attribution bias that affects any study examining long term mortality using ICD coding. Coding of deaths may be effected by autopsy rates and the accuracy of CHD coding outside hospital. The 1995 United Kingdom Heart Attack Study suggested that up to age 65 death certification and coding were very accurate (within 4%), however, for deaths occurring above age 65, an over-estimate of about 20% was likely [13]. Coupled with the continuous process of improving the accuracy of certification and coding over the last three decades, it is likely that the apparent fall in CHD death rates in older groups has been slightly over-estimated [14]. While this adds potential variance to the mortality rates presented in this paper the falls in mortality rates themselves are real, just not quite as big in the elderly as perceived [15,16].

### Comparison with existing literature

Age-standardised CHD mortality rates for both men and women calculated using the coding frame described in Table 1 (results not shown here) show good agreement with Office for National Statistics (ONS) [1]. It has been suggested that pre-1967 definitions of CHD were inconsistent due to the coding of some CHD deaths as 'other myocardial degeneration' (ICD codes 422 in ICD – 6 and ICD – 7). It has proved difficult to separate out other causes of death and previous work has used definitions which exclude code 422 in ICD-6 and ICD-7 [1]. For this reason, the ONS results rely on two calculations for all pre-1967 rates, one including 'other myocardial degeneration' and one excluding it. The data presented here do not include 'other myocardial degeneration', and the 1950 to 1967 age-standardised mortality rates are very similar to those calculated by the ONS. A difficulty may arise when interpreting birth cohort results by age at death because the effect of revisions of ICD coding will affect different cohorts at different ages.

Previous work supports our finding that CHD mortality rates appear to be levelling out and perhaps reversing in younger age groups. Wilson and Siskind [4] studied death registrations for CHD using 5-year sex and age specific birth cohorts and found that in the youngest male cohorts (1950–54, deaths at ages 25 – 29) there was evidence of a flattening in rates. More recently a study set in the U.S. [5] described a reversal in previously declining CHD death rates. These authors identified an annual 1.3% increase in CHD mortality among women aged 35 to 44 between 1997 and 2002.

## Conclusion

Although CHD mortality rates continue to drop in older age groups the actual burden of coronary heart disease is increasing due to the ageing of the population. The rate of improvement in CHD mortality appears to be beginning to decline and maybe even reversing among younger women. CHD mortality rates of those younger than 60 are worse for those born in the early 20^th ^century than for those born in the 19^th^. If this trend in younger age groups is not halted the burden of coronary heart disease is likely to increase.

## Competing interests

The authors declare that they have no competing interests.

## Authors' contributions

SC, MOF, SA and PS contributed to the conception and design of the project and the interpretation of data. SA and PS acquired the data for the project and conducted the analysis. All authors have been involved in the drafting and revision of the MS. All authors have approved this version of the MS.

## Pre-publication history

The pre-publication history for this paper can be accessed here:


